# The cyclin D1b splice variant: an old oncogene learns new tricks

**DOI:** 10.1186/1747-1028-1-15

**Published:** 2006-07-24

**Authors:** Karen E Knudsen

**Affiliations:** 1 Department of Cell Biology, Center for Environmental Genetics, and UC Cancer Center, University of Cincinnati College of Medicine, Cincinnati, Ohio 45267-0521, USA

## Abstract

The function of cyclin D1 as a positive regulator of the cell cycle and proto-oncogene has been well established. Cyclin D1 elicits its pro-proliferative function early in G1 phase, through its ability to activate cyclin dependent kinase (CDK) 4 or 6. Active CDK4/6-cyclin D1 complexes phosphorylate substrates that are critical for modulating G1 to S phase progression, and in this manner promote cellular proliferation. Emerging data from a number of model systems revealed that cyclin D1 also holds multiple, kinase-independent cellular functions. First, cyclin D1 assists in sequestering CDK inhibitors (e.g. p27^kip1^), thus bolstering late G1 CDK activity. Second, cyclin D1 is known to bind and modulate the action of several transcription factors that hold significance in human cancers. Thus, cyclin D1 impinges on several distinct pathways that govern cancer cell proliferation. Although intragenic somatic mutation of cyclin D1 in human disease is rare, cyclin D1 gene translocation, amplification and/or overexpression are frequent events in selected tumor types. Additionally, a polymorphism in the cyclin D1 locus that may affect splicing has been implicated in increased cancer risk or poor outcome. Recent functional analyses of an established cyclin D1 splice variant, cyclin D1b, revealed that the cyclin D1b isoform harbors unique activities in cancer cells. Here, we review the literature implicating cyclin D1b as a mediator of aberrant cellular proliferation in cancer. The differential roles of cyclin D1 and the cyclin D1b splice variant in prostate cancer will be also be addressed, wherein divergent functions have been linked to altered proliferative control.

## Background

Cyclin D1 is a focal point for integrating mitogenic stimulation with cellular proliferation [1,2]. Mitogenic signals typically induce increases in cyclin D1 mRNA expression and translation, thereby increasing the cellular pool of the protein product. The pro-proliferative function of cyclin D1 is mediated through its ability to regulate the cell cycle machinery, and excessive cyclin D1 expression and/or activity is a hallmark of several tumor types [3,4]. In addition, cyclin D1 has cell cycle-independent functions through its ability to modulate transcription factor action [5]. Given the importance of cyclin D1 in human disease, concerted effort has been directed at delineating the mechanisms by which cyclin D1 is dysregulated in cancer. In selected tumor types, cyclin D1 is overexpressed as a result of chromosomal translocation or amplification of the cyclin D1 (CCND1, PRAD) locus [1,6]. Intragenic somatic mutations of cyclin D1 are rare, but a polymorphism of cyclin D1 that occurs in a splice donor site has been epidemiologically linked to increased cancer risk or poor prognosis in a number of tumor types (reviewed in [7]). Recent functional analyses have revealed that the protein product of an alternately spliced transcript, cyclin D1b, harbors overlapping but distinct functions as compared to full length cyclin D1. Herein, the potentially unique functions of cyclin D1b in cancer are discussed, with an eye to the future for addressing the mechanism and consequence of cyclin D1b expression in human disease.

### Mitogen regulation of cyclin D1 expression and stability

Given the importance of cyclin D1 in modulating cellular proliferation, it is not surprising that cyclin D1 expression is stringently regulated [1,3]. Growth factor stimulation typically activates cyclin D1 mRNA production, as can be achieved through direct induction of the cyclin D1 promoter [8,9]. Several sequence specific transcription factors have been shown to modulate cyclin D1 promoter activity in response to mitogens, including peptide growth factors and steroidal hormones [10]. However, the molecular effectors of cyclin D1 locus modulation can be tissue specific. For example, in mammary epithelial or breast cancer cells, cyclin D1 mRNA expression can be regulated by estrogen receptor alpha (ER) binding to regulatory regions within the cyclin D1 locus. It was initially determined that ER tethers to Sp1 in the proximal cyclin D1 locus to stimulate cyclin D1 mRNA production [11,12]. However, recent analyses revealed that the ability of estrogen to induce cyclin D1 in breast cancer cells can be executed though an enhancer region downstream of the cyclin D1 coding region, and involves recruitment of ERβ to that locus (M. Brown, personal communication). By contrast, in colon cancer cells, the Wnt pathway is a key determinant of cyclin D1 expression, and this action is mediated through β-catenin/TCF [13]. Thus, cyclin D1 mRNA accumulation is coordinated by distinct transcription factors, dependent on differential mitogen function and tissue specific cellular milieu.

Once produced, the cyclin D1 transcript is subjected to multiple modes of post-transcriptional control, including regulation at the level of mRNA stability, mRNA translation, subcellular protein localization, and targeted protein degradation [1,3,14]. Stability of the cyclin D1 transcript is regulated through 3' UTR regions and/or through association with RNA binding proteins HuR and AUF1 [15-17]. Transcript destabilization occurs through activation of divergent signaling agents (e.g. prostaglandins and rapamycin) [18,19], whereas oncogenic stimuli such as Wnt/β-catenin stabilize the cyclin D1 mRNA [20]. The transcript is also subjected to translational control. For example, PKC-alpha represses cellular proliferation in part through its ability to block cap-dependent initiation of cyclin D1 translation in intestinal epithelia [21]. In many tumor types, the eukaryotic translation initiation factor eIP4e is dysregulated, and functions to induce cyclin D1 translational efficiency [22]. Thus, regulation of the cyclin D1 transcript and subsequent protein synthesis represent major modes of proliferative control.

Ultimately, the cyclin D1 protein is translocated into the nucleus to exert its pro-proliferative function in G1 phase of the cell cycle [14]. Subsequently, cyclin D1 is phosphorylated on threonine 286; this event facilitates CRM1 recognition of the phosphoprotein, and subsequent nuclear export and degradation [23]. The importance of nuclear export control for appropriate cellular proliferation is evident, in that cyclin D1 mutants incapable of thr286 phosphorylation and nuclear export have markedly enhanced oncogenic potential, and transgenic expression induces B-cell lymphoma in murine models [23,24]. Somatic mutations of this residue have been identified in endometrial and esophageal cancers, thus indicating that disruption of cyclin D1 nuclear export may participate in tumor development and/or progression in this tissue [25,26]. As with many of the cyclins, regulated degradation also governs cyclin D1 action. The C-terminus of cyclin D1 contains a PEST domain, which was hypothesized to act as a degradation motif, but the contribution of this motif for cyclin D1 destruction has yet to be rigorously demonstrated. Although the mechanisms remain elusive, ubiquitin-mediated cyclin D1 degradation can be induced by a myriad of anti-mitogenic signals, including DNA damage [27,28]. Interestingly, DNA damage-mediated cyclin D1 destruction can occur in the nucleus, indicating that nuclear export is not always required for cyclin D1 turnover [27]. Combined, these observations indicate that the cyclin D1 protein is stringently controlled, wherein mitogens generally act to induce and/or stabilize the protein, and anti-mitogenic signals dampen cyclin D1 accumulation.

### Cyclin D1b: the product of an alternate splicing event

**Figure 1:**
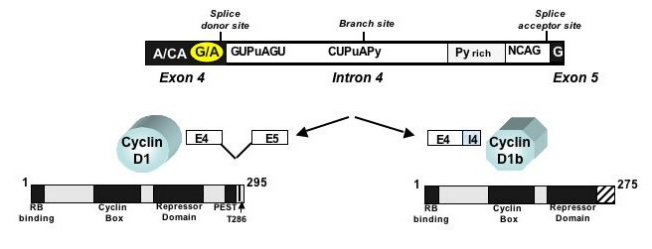
**The cyclin D1 transcript is subject to alternative splicing**. Alternative splicing is known to occur at the exon4/intron 4 boundary. Effective splicing at this junction results in the well-characterized cyclin D1 product (bottom left), which contains discrete motifs that regulate cell cycle control, subcellular localization, and transcriptional regulation. Failure to splice at the exon4/intron 4 boundary results in the cyclin D1b protein product, which harbors a divergent C-terminus (hatched region). The G/A870 polymorphism (circled) is thought to influence the splicing event.

Cyclin D1 is encoded by 5 exons, and harbors distinct motifs that exert its biological function (Figure 1). In 1995, Betticher et al. described a cyclin D1 variant, now termed cyclin D1b, which results from alternate splicing at the exon 4, intron 4 boundary [29]. The location of the alternate splicing event generated significant interest, as the splice donor site encompasses a known polymorphism of cyclin D1 that has been associated with increased cancer risk and/or poor prognosis in a multitude of tumor types. The polymorphic residue (G/A870) is silent for amino acid sequence, but the "A" allele was hypothesized to reduce the efficacy of the splice donor site and favor production of an alternate transcript encoding cyclin D1b. The relationship of the G/A870 polymorphism to the production of cyclin D1b has been recently reviewed [7], and conservative assessment of the established data reveals that while the A/A genotype may predispose to cyclin D1b production, additional factors contribute to the production of this variant. Most recently, SWI/SNF chromatin remodeling enzymes have been implicated in modifying splicing of the cyclin D1 mRNA [30]. These observations indicate that cyclin D1 splicing may represent another dynamic mechanism to regulate cyclin D1 function, and further investigation is needed to reveal the cadre of variables that impinge on the splicing event.

Failure to splice at the exon 4/intron 4 boundary results in complete loss of exon 5 encoded sequences and the acquisition of a novel 33 amino acid stretch at the C-terminal end, as contributed by translation into intron 4 and the presence of an intronic stop codon. It is notable that cyclin D1b lacks both the C-terminal PEST sequence and thr286 residue; lack of these domains predict that the protein would reside in the nucleus and be intrinsically more stable than full-length cyclin D1. Interrogation of these hypotheses validated the first prediction, in that immunostaining for cyclin D1b or the use of GFP-tagged cyclin D1b revealed the protein to be largely nuclear in localization [31,32]. These preliminary observations underscore the need to carefully evaluate cyclin D1 expression in tumors, taking into account that many available antisera fail to recognize the cyclin D1b isoform. Moreover, consideration of cellular compartment may be of importance, as several studies have shown that cyclin D1 staining in selected tumor types can be restricted to the cytoplasm ([33-35], and Comstock and Knudsen, in preparation). Immunohistochemical analyses of cyclin D1 relevance for prognosis or outcome may need to be re-evaluated for relative isoform expression and impact dependent on these parameters.

The second prediction based on cyclin D1b composition proved incorrect, as cyclin D1b was not significantly more stable than cyclin D1; rather, half-lives of the two proteins were similar between the two proteins [31,32]. Therefore, the inability of cyclin D1b to utilize thr286-driven nuclear export may not preclude regulated protein degradation. Interestingly, the nuclear dynamics of the two cyclin D1 isoforms were quite distinct when monitored in living cells [32]. Full-length cyclin D1 was relatively immobile, thus indicating that in addition to nuclear export, cyclin D1 may be regulated by retention into an immobile nuclear fraction. By contrast, two distinct pools of cyclin D1b were observed in the nucleus of living cells. The first population behaved in a manner analogous to cyclin D1, wherein the protein was largely immobile. The second population (which constituted approximately one-third of the total cyclin D1b) showed markedly enhanced nuclear mobility. Further analyses revealed that cyclin D1b is highly mobilized in S-phase, thus indicating a distinct mode of regulation as compared to cyclin D1. The relative contribution of the mobile versus immobile fraction of cyclin D1b for function has yet to be discerned.

Although many of the functional domains of cyclin D1 are retained within cyclin D1b, subsequent analyses revealed that cyclin D1b harbors markedly increased oncogenic function. This unexpected phenomenon was first observed in NIH3T3 cells, wherein cyclin D1b but not cyclin D1 was sufficient to drive cellular transformation *in vitro *(as monitored by focus formation assays) or tumor formation *in vivo *[31,32]. Similarly, cyclin D1 null murine embryo fibroblasts acquired anchorage independence after restoration of cyclin D1b but not full-length cyclin D1 [36]. These collective observations suggested that cyclin D1b has enhanced oncogenic potential. Cyclin D1b has been shown to be expressed in a multitude of cancer cell types (including mantle cell lymphoma cell lines, esophageal cancers, B-lymphoid malignancies, prostate cancer, colon cancer, and breast cancer) [31,37-39], indicating that this heightened oncogenicity may facilitate tumorigenesis. Interestingly, a sampling of primary mantle cell lymphomas revealed that the cyclin D1b transcript did not result in detectable protein production [40], thus revealing that the cyclin D1b mRNA or protein may be under strict regulation in this tumor type. Given the potency of cyclin D1b in inducing cellular transformation and tumor formation, concerted effort has been recently directed at delineating the underlying mechanisms of its oncogenicity. As discussed hereafter, differential cyclin D1b functions have been identified for both the cell cycle and transcriptional regulatory functions normally associated with cyclin D1.

### CDK dependent cell cycle regulation

**Figure 2:**
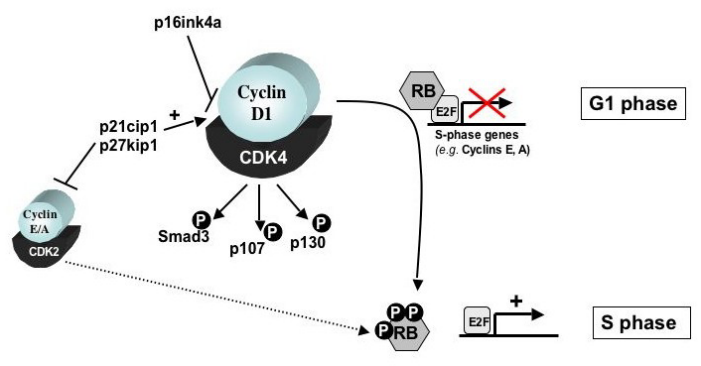
Cell cycle functions of cyclin D1.

The role of full-length cyclin D1 as a critical effector of G1-S progression is well established, and these functions of cyclin D1 are manifest through modulation of the cell cycle machinery [1,3,4]. Once produced, cyclin D1 binds to either cyclin dependent kinase 4 (CDK4) or CDK6, dependent on cell type, and serves to activate the kinase moiety (Figure 2). Active cyclin D1-CDK4/6 complexes phosphorylate a rather limited set of substrates whose activities play pivotal roles in controlling G1-S progression [4]. The most well-defined substrate of cyclin D1-CDK4/6 complexes is the retinoblastoma tumor suppressor protein (RB) [41-43]. In its hypophosphorylated state, RB exerts potent anti-proliferative action through its ability to form transcriptional repressor complexes on the promoters of genes required for S-phase progression [44,2,45]. CDK mediated phosphorylation of RB relieves its transcriptional repressor function, and thus promotes a permissive state for cell cycle progression. The importance of cyclin D1-CDK4/6 in this process was exemplified in that RB null cells are refractory to the proliferative action of the cyclin D1-CDK4 complex, and RB deficient tumor cells often exhibit reduced cyclin D1 dependency [9,46-48]. Finally, it was shown that the p16ink4a tumor suppressor, which inhibits cellular proliferation through its ability to displace cyclin D1 from the CDK4 complex, is ineffective in RB null cells [46,49,50]. Together, these data indicated that RB is the principal substrate utilized by full-length cyclin D1-CDK4/6 to modulate G1-S control, and put forward the hypothesis that the "p16ink4a-cyclin D1-RB axis" is a key regulator of the mitotic cell cycle. This hypothesis is borne out in the analyses of human tumors, wherein over 90% of human tumors show p16ink4a loss, loss of RB, excessive expression of cyclin D1, or activating mutation of CDK4 [6]. These aberrations are often tissue specific, thus indicating that different tumor types favor differential avenues to disrupt the p16-cyclin D1-RB axis. Recent reports indicate that cyclin D1-CDK4/6 complexes phosphorylate additional substrates, including Smad3, p107, and p130, each of which impinge on cellular proliferation [51,41,52]. Combined, it is hypothesized that the ability of full-length cyclin D1 to activate CDK4/6 kinase function is a major mechanism of cell cycle control.

Comparative analyses of cyclin D1b effects on CDK4/6 function and cell cycle proliferation have revealed unexpected disparities in function. While cyclin D1b is efficient in CDK4 binding and can induce CDK4 activity *in vitro*, it is poorly effective at mediating Rb phosphorylation in osteosarcoma cells [31,32]. Consistent with this observation, cyclin D1b was markedly impaired in its ability to relieve RB-mediated transcriptional repression at the cyclin A promoter, a key transcriptional target through which RB governs cell cycle progression [32]. These findings likely underlie the concurrent observation that cyclin D1b is significantly compromised in its ability to overcome RB-mediated cell cycle arrest and induce cell cycle progression, as compared to full-length cyclin D1. Therefore, although cyclin D1b harbors heightened oncogenic function, the protein variant appears compromised in its ability to elicit RB kinase activity and activate cellular proliferation. It will be of interest to determine whether the consequence of cyclin D1b expression is dependent on cell context, as the number of model systems utilized to assay distinctions between cyclin D1 and cyclin D1b are limited. Moreover, the relative stoichiometry of each variant in specific cell types may differentially impinge on cyclin D1b activity, and this parameter should be carefully considered. For selected tumor types, cyclin D1 has been postulated as a potential target for therapeutic intervention (e.g. pancreatic and breast cancer) [53-55], and how the disparate functions of cyclin D1b may influence outcome should likely be incorporated as a major consideration. The unique mechanisms underlying cyclin D1b mediated transformation potential are actively under investigation, and are likely to include non-canonical effects on the cell cycle machinery as well as cell cycle independent functions.

### CDK4 kinase-independent cell cycle regulation

It is now appreciated that full-length cyclin D1 affects control over the cell cycle machinery using both kinase-dependent and kinase-independent mechanisms. For example, cyclin D1 acts through sequestration of the CDK inhibitors p21^cip1 ^and p27^kip1 ^to govern G1-S progression [49,56]. This class of CDK inhibitor predominantly blocks CDK2 activity, but paradoxically promotes association (and thus activation) of cyclin D1-CDK4/6 complexes [57]. The interaction of p21^cip1 ^and p27^kip1 ^with the complex is mediated through direct association with cyclin D1; thus cyclin D1 indirectly promotes CDK2 activity by acting as a sink for p21^cip1 ^and p27^kip1^. Conversely, binding of p16ink4a to cyclin D1 displaces p21^cip1 ^or p27^kip1 ^and renders these CDK inhibitors free to bind and inhibit CDK2 activity. Through these mechanisms, full-length cyclin D1 utilizes both CDK dependent and independent functions to modulate cell cycle control.

Present studies have yet to address the ability of the cyclin D1b variant to modulate p21^cip1 ^or p27^kip1 ^activity. The domain of cyclin D1 that associates with p21^cip1 ^has been identified (amino acids 20–170) [58], and is retained in cyclin D1b. It is thereby predicted that cyclin D1b would maintain the ability to sequester p21^cip1 ^or p27^kip1 ^from CDK2 complexes. p21^cip1 ^is also known to promote cyclin D1 nuclear localization through its ability to inhibit GSK-3β phosphorylation of thr286 [57], but this function holds no consequence for the cyclin D1b variant. Once the mechanisms of cyclin D1b-mediated transformation potential are identified, it will be essential to assess the impact of CDK inhibitor function on this property.

### Cyclin D1 mediated transcriptional control

In the late 1990's an unexpected role for cyclin D1 was uncovered, as it was established that cyclin D1 interacts with and modulates a number of transcription factors whose actions are involved in human cancers (reviewed in [5]). In general, these functions of cyclin D1 occur through CDK-independent mechanisms and are manifest through direct transcription factor binding. One of the first transcription factors found to associate with cyclin D1 was estrogen receptor (ER) alpha [59,60], which serves a critical role in breast cancer development and management. These early studies demonstrated that cyclin D1 utilizes an intrinsic "LxxLL" (nuclear receptor association) motif to enhance ER function, and promote recruitment of the SRC1 and/or P/CAF co-activators [61,62]. Cyclin D1 also blocks the ability of BRCA1 to repress ER activity [63], providing an indirect mechanism of ER activation. These observations suggested the existence of a positive feedback loop to control breast cancer cell proliferation, as ER binds to the cyclin D1 locus and induces cyclin D1 expression [64]. Thus, in breast cancer cells it was proposed that cyclin D1 could use both CDK dependent mechanisms and CDK independent mechanisms to promote proliferation. Mice deficient in cyclin D1 but rescued with the cyclin D1-KE mutant (defective in CDK4 activation) have normal mammary gland development, whereas the cyclin D1 null animals are blunted in this process [65]. These data demonstrate that CDK dependent functions of cyclin D1 are apparently not required for proliferation and development of murine mammary epithelia. However, both the cyclin D1 nulls and the cyclin D1-KE mice are unable to support Her2/Neu induced mammary tumor formation, indicating that CDK4 activity is required for this oncogenic event in mice [65]. In human breast cancers, gene expression analyses of several hundred specimens showed that transcriptional modulatory function of cyclin D1 may play an influential role in this tumor type [66]. Intriguingly, cyclin D1 positive breast cancers typically exhibit low proliferative rates, and dual positive immunostaining for ER and cyclin D1 expression has been associated with positive outcome and/or survival in some studies [67-70], suggesting that the ability of cyclin D1 to promote ER activity may enhance a differentiative phenotype. Thus, while the relative impact of cyclin D1 mediated transcriptional control versus CDK4 modulation in breast cancer awaits further investigation, an abundance of observations support a role for cyclin D1 in transcriptional control for human tumors.

The ER-modulatory function of cyclin D1 is likely disrupted in the cyclin D1b variant, which lacks the LxxLL motif required to modulate the receptor. Preliminary studies support this concept, wherein cyclin D1b induction yields no significant alteration of ER function (Burd and Knudsen, unpublished observations). The impact of this loss of function on breast tumorigenesis or tumor growth has yet to be examined. However, this variant is known to be expressed in breast cancer cells [39], and the polymorphism that may predispose to cyclin D1b production has been associated in some (but not all) studies with increased cancer risk [7,71]. Given the potential link between dual ER/cyclin D1 expression and outcome, this parameter may be of significance.

Since the discovery that cyclin D1 can modulate transcription factor function, several factors have been established as targets of full-length cyclin D1 modulation, independent of CDK function. These factors include STAT3, v-myb, DMP1, BETA2/Neuro D, and C/EBPβ [66,72-76]. Remarkably, the largest class of cyclin D1 mediated transcription factors is the nuclear receptor superfamily, including ER, the thyroid hormone receptor (TR), peroxisome proliferator gamma (PPARγ), and the androgen receptor (AR). However, the mechanisms and outcome of cyclin D1 action are disparate for the different nuclear receptors. While cyclin D1 activates ER through its LxxLL motif [61], cyclin D1 serves as a repressor of TR, PPARγ, and AR function. The mechanisms utilized to modulate nuclear receptor function are distinct. For TR it was shown that cyclin D1 associates directly with histone deacetylase (HDAC) 3 but not HDAC1, and recruits this co-repressor to the TR complex [77]. Some overlap is observed with PPARγ, wherein cyclin D1 utilizes a defined motif resembling a helix-loop-helix domain to interact with and block both ligand-dependent PPARγ activity and PPARγ expression itself [78,79]. The biological consequence of the cyclin D1-PPARγ association was validated, in that PPARγ-mediated adipocyte differentiation was enhanced in cyclin D1 -/- fibroblasts, and deletion of cyclin D1 induced PPARγ expression and activity. The concept that cyclin D1 regulates PPARγ function has additional implications for breast cancer, wherein PPARγ is known to promote breast epithelial cell differentiation, and histological analyses of breast cancer specimens revealed that PPARγ is reduced in tumors with a high cyclin D1 index [78,80]. Thus, the ability of cyclin D1 to antagonize PPARγ may alter the potential anti-proliferative action of PPARγ ligands in this tissue type. Interestingly, the mechanism of cyclin D1-mediated PPARγ regulation was attributed to HDAC1 and HDAC3 recruitment [79]. CDK-dependent mechanisms may also influence nuclear receptor activity, as active cyclin D1-CDK4 activity is known to cause sequestration of the GRIP1 nuclear receptor co-activator into inactive, sub-nuclear structures [81]. Thus, while multiple mechanisms are implicated as effectors of cyclin D1 function, these may be nuclear receptor-specific.

Analysis of cyclin D1b expression patterns and consequence for nuclear receptor activity has yet to be fully established for TR or PPARγ, although it has been shown that cyclin D1b can repress TR function in transient assays [37]. The functional domains required for interaction with both receptors is retained, thus identifying these receptors as putative targets of cyclin D1b function in specific tissues. For AR, it has been established that cyclin D1b is significantly altered in its ability to modulate receptor function, and as discussed below, this disparate action has consequence in prostate cancer cells [37].

### Cyclin D1 and cyclin D1b effects on AR activity in prostate cancer cells

Prostate cancer cells are unique amongst tumors types in that they are dependent on AR function for growth and survival [82]. The androgen dependence of prostate cancer cells is exploited in treatment of disseminated prostate cancers, wherein ablation of AR activity (through either ligand depletion and/or through the use of AR antagonists) is the first line of therapeutic intervention [83,82,86]. These therapies are initially effective, and induce a mixed response of cell cycle arrest or apoptosis of the prostate cancer cells, regardless of whether or not the tumor remains organ-confined. However, recurrent tumors form within a median time of 2–3 years, and no effective treatment has been identified to treat recurrent disease. Interestingly, analyses of recurrent tumors revealed that in the great majority of therapy-resistant tumors, AR has been re-activated, as achieved through either AR amplification, AR mutation, ligand-dependent AR activation, and/or through excessive production of AR co-activators [82]. Moreover, deregulation of AR activity has been validated in tumor models to be sufficient for therapeutic bypass [87,88]. Together, these observations indicate that AR is a critical determinant for prostate cancer growth and progression.

**Figure 3:**
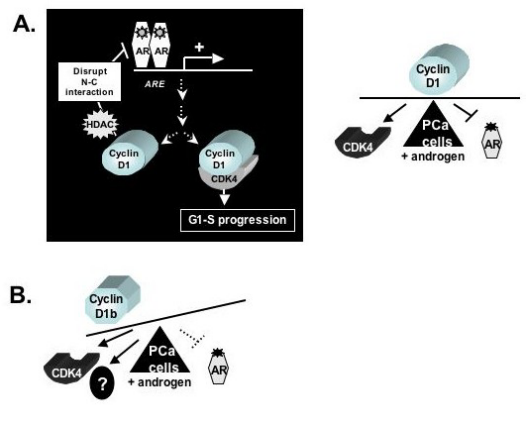
**D-type cyclins regulate androgen receptor function in prostate cancer cells**. **A**) Cyclin D1 is induced following androgen stimulation, and acts through discrete mechanisms to both promote cell cycle progression and attenuate androgen receptor (AR) activity (left panel). These control mechanisms are hypothesized to modulate the strength and duration of the androgen response (right panel). **B**) The cyclin D1b variant harbors enhanced oncogenic function as compared to cyclin D1, and interrogation of cell cycle function suggests that cyclin D1b may impinge on additional factors to promote proliferation. In the context of prostate cancer, compromised ability of cyclin D1b to modulate AR function may disrupt modulation of the androgen response, and yield an enhanced proliferative response.

AR stimulates prostate cancer cell growth through its ability to stimulate gene expression [89,90]. Although the proximal targets that elicit this mitogenic response have yet to be identified, AR activation results in accumulation of D-type cyclins, including both cyclin D1 and cyclin D3 [91]. Functional studies revealed that induction of D-type cyclins resulted in enhanced CDK4 activity and RB phosphorylation, as expected based on the ability of androgen to induce G1-S progression. Forced expression of full-length cyclin D1 (and/or CDK4) under conditions of androgen ablation failed to bypass cell cycle arrest, whereas viral oncoprotein induced RB inactivation strongly promoted androgen-dependent cellular proliferation, thus indicating that cyclin D1 is not sufficient to induce cell cycle progression in this cell type [91,92]. By contrast, studies performed in the presence of androgen indicated that accumulated cyclin D1 markedly attenuates cellular proliferation in this cell type, dependent on the ability of cyclin D1 to modulate AR function [93]. Thus, a feedback model has been proposed, wherein cyclin D1 elicits a pro-proliferative, CDK4 dependent function to stimulate cell cycle progression after androgen stimulation, and accumulated cyclin D1 serves to modulate the strength and duration of the androgen response through attenuating AR function (Figure 3A). It is notable that unlike many of the other nuclear receptors (e.g. ER) the AR is not degraded after ligand stimulation, but is actually stabilized by ligand binding [94]. Thus, this intrinsic mechanism of feedback control may be important for titering the mitogenic response. Strikingly, the cyclin D1b variant is compromised for this feedback control mechanism, and this disparate action is attributed to its altered ability to regulate AR function.

Full-length cyclin D1 utilizes multiple mechanisms to modulate AR activity, and these functions have been well described. First, it has been shown that the principal cyclin D1 binding site resides in an N-terminal region of the AR that modulates ligand-dependent conformational changes (the ^23^FxxLF^27^) motif [95]. In response to ligand, the FxxLF motif docks into a C-terminal, hydrophobic cleft of the receptor, and this "N-C terminal interaction" is important for both AR activity and chromatin association. Cyclin D1 binding strongly reduces N-C interaction, and this passive mechanism underlies a significant portion of cyclin D1 repressor function. Second, cyclin D1 employs HDACs to modulate AR activity, and cyclin D1 repressor function is reversed through HDAC inhibition [93]. Cyclin D1 associates with HDAC3 in prostate cancer cells, through a repressor domain motif that is required and sufficient for AR modulation [96]. Lastly, it has been proposed that cyclin D1 can compete for P/CAF binding to AR, thus precluding co-activator function [97]. However, this observation was not confirmed in all model systems analyzed [93]. The net result of these actions is down-regulation of AR function, as demonstrated by slowed cell cycle progression and down-regulation of endogenous PSA (prostate specific antigen) expression, an AR target gene utilized clinically to monitor prostate cancer growth and recurrence [98]. The repressor functions occur at stoichiometric levels of AR and cyclin D1, thus indicating that modest elevations of cyclin D1 can have significant effects on prostate cancer growth.

Cyclin D1b maintains the repressor domain required for each of these cyclin D1 functions and indeed, the ability to block ligand induced N-C interaction and to recruit HDACs to the AR complex is generally conserved in the cyclin D1b variant [37]. However, functional analyses revealed that cyclin D1b is selectively compromised for AR regulation, dependent on the target gene analyzed. Compared to full-length cyclin D1, the cyclin D1b variant was significantly deficient in its ability to repress AR-dependent expression of the prostate specific antigen (PSA) mRNA expression in prostate cancer cells, a target gene used clinically to monitor prostate growth and progression. Similar effects were observed in the analyses of other AR target genes (e.g. Slp, sex-limited protein), whereas no distinctions were observed using the non-AR specific MMTV locus. The most striking disparity in function between the two cyclin D1 proteins in the context of prostate cancer was observed using cellular proliferation as an endpoint. Consistent with the negative feedback model, full-length cyclin D1 markedly inhibited cell cycle progression in AR dependent (but not AR negative) prostate cancer cells. However, cyclin D1b was completely ineffective at attenuating the proliferative response, and in fact stimulated cellular proliferation in the AR positive cells. Collective analyses of cyclin D1b mediated AR regulation indicated that the altered AR regulatory capacity of cyclin D1b at least partially underpins its ability to induce cell cycle progression in this cell type (Figure 3B), and likely acts in concert with the heightened oncogenic capacity of cyclin D1b revealed in NIH3T3 studies. These data suggest that cyclin D1b may hold multiple oncogenic functions in prostate cancer cells, and predict that the reliance of this tumor cell type on AR function could result in selective pressure to produce the cyclin D1b isoform. Whether these predictions hold true will require deeper consideration of cyclin D1 isoform expression and/or function during prostate cancer development and progression.

### Cyclin D1b and prostate cancer

To date, few studies have assessed the role of cyclin D1 in prostate cancer. While cyclin D1 is frequently amplified and/or overexpressed in breast cancer [71], cyclin D1 overexpression is rarely observed in prostatic tumors [99]. When excessive cyclin D1 has been reported, this parameter has no significant independent prognostic value, and is predictive of poor prognosis only in the presence of high cyclin A (thus indicative of a high proliferative index) [99]. In a comparative study of local disease, few tumors showed cyclin D1 expression (11% of tumors showed greater than 20% positive nuclei), whereas bone metastases showed increased frequency of cyclin D1 expression (68%), thus indicating that the role of cyclin D1 may be altered in distant metastases [100]. Still, no correlation with pathological grade or recurrent tumor formation was observed in these data sets. In animal models, cyclin D1 is downregulated or sequestered in the cytoplasm of advanced tumors, thus indicating that the AR regulatory function may be lost in this disease [101]. Recent reports indicate that knockdown of AR expression (via siRNA) results in induction of cyclin D1 expression [102], thus indicating that the pressure to restrain cyclin D1 expression may be lifted upon AR elimination. These collective data indicate that the role of cyclin D1 in prostate cancer is complex, and that alterations in full-length cyclin D1 expression and localization may have both positive and negative effects on tumor growth.

Recently it was revealed that the cyclin D1b variant is highly expressed in localized prostate cancer, as compared to matched normal tissue [37]. Lymph node metastases of prostate cancer also maintained cyclin D1b expression. Given the relative poor efficacy of cyclin D1b in titering AR function, high cyclin D1b expression may render a mechanism whereby prostate cancer cells may promote CDK4 activity (and/or other pro-proliferative targets utilized by cyclin D1b) while concurrently evading mechanisms to modulate the AR dependent growth response. Based on this hypothesis, one might speculate that specific contextual variables (e.g. AR expression and dependence) may distinguish cyclin D1b action in individual cell types (e.g. prostate cancer cells versus NIH3T3 cells). Identification of these potential variables will afford a deeper understanding of cyclin D1b oncogenic potential and indeed are requisite to clarify the biological consequence of cyclin D1b in individual tumor types. Although the sample size examined was small, preliminary data demonstrated that cyclin D1b was also highly expressed in prostatic intraepithelial neoplasias (PIN lesions), which are thought to be precursors of adenocarcinoma. These observations suggest that cyclin D1b expression may be induced early in tumor development, and may participate in the transformation of prostatic epithelial cells. Additional investigation in this tumor type will be needed to segregate the relative impact of transcriptional versus cell cycle and/or oncogenic effects of cyclin D1b on tumor growth and progression.

### Conclusions and future considerations

The cyclin D1b variant exhibits unique properties that may underlie its enhanced oncogenic potential. First, cyclin D1b is a constitutively nuclear protein, and has enhanced transformation capacity in tested model systems. Second, cyclin D1b is a poor catalyst of CDK4 activity in cell systems analyzed to date, thus suggesting that cyclin D1b utilizes non-canonical mechanisms to induce its oncogenic function. Third, cyclin D1b retains the ability of full-length cyclin D1 to modulate the action of some (but not all) transcription factors, and this differential function may hold consequence in selected tumor types. In prostate cancer, the disparate ability of cyclin D1b to govern AR function appears to hold significance for both the control of transcriptional regulation and cellular proliferation.

Despite these advances in delineating cyclin D1b function, many critical questions remain to be addressed. *First, what are the factors that govern cyclin D1b production? *A very limited set of variables have been shown to influence the aberrant splicing event (e.g. the G/A870 polymorphism, SWI/SNF function), but it is clear that additional factors must assist in controlling the splicing decision. *Second, what function(s) of cyclin D1b underlie its enhanced transforming potential? *Possibilities to be considered include novel protein associations, novel substrate recognition, unexpected effects of sustained nuclear localization, and/or loss of responsiveness to anti-mitogenic signals. *Third, what is the relative impact of cyclin D1b on transcriptional regulation*? Early indications suggest that in hormone dependent cancers that cyclin D1b transcriptional effects are distinct, but the tissue specific effects of cyclin D1b on gene expression have been remarkably understudied. *Fourth, what is the overall biological consequence of cyclin D1b expression in cancer? *The impact of cyclin D1b is likely to be cell type specific, subject to relative stoichiometry with full-length cyclin D1, and dependent on the availability of cofactors to mitigate cellular response. Tissue specific distribution, relative patterns of expression in tumor and matched normal tissue should also be considered, so as to sort out the timing and impact of cyclin D1b production. In sum, analyses of cyclin D1b expression in a multitude of tumor types underscores the potential importance of cyclin D1b for tumor development and/or progression, and rigorous investigation of this novel oncogene is necessary to reveal the divergent mechanisms by which cyclin D1b elicits its potent effect in tumor cells.
